# Safety Profile of the Trastuzumab-Based ADCs: Analysis of Real-World Data Registered in EudraVigilance

**DOI:** 10.3390/biomedicines12050953

**Published:** 2024-04-25

**Authors:** Claudiu Morgovan, Carmen Maximiliana Dobrea, Anca Butuca, Anca Maria Arseniu, Adina Frum, Luca Liviu Rus, Adriana Aurelia Chis, Anca Maria Juncan, Felicia Gabriela Gligor, Cecilia Georgescu, Steliana Ghibu, Andreea Loredana Vonica-Tincu

**Affiliations:** 1 Preclinical Department, Faculty of Medicine, “Lucian Blaga” University of Sibiu, 550169 Sibiu, Romania; claudiu.morgovan@ulbsibiu.ro (C.M.); anca.butuca@ulbsibiu.ro (A.B.); anca.arseniu@ulbsibiu.ro (A.M.A.); adina.frum@ulbsibiu.ro (A.F.); liviu.rus@ulbsibiu.ro (L.L.R.); adriana.chis@ulbsibiu.ro (A.A.C.); ancamaria.juncan@ulbsibiu.ro (A.M.J.); felicia.gligor@ulbsibiu.ro (F.G.G.); loredana.vonica@ulbsibiu.ro (A.L.V.-T.); 2 Faculty of Agriculture Science, Food Industry and Environmental Protection, “Lucian Blaga” University of Sibiu, 550012 Sibiu, Romania; cecilia.georgescu@ulbsibiu.ro; 3 Department of Pharmacology, Physiology and Pathophysiology, Faculty of Pharmacy, “Iuliu Hatieganu” University of Medicine and Pharmacy, 400012 Cluj-Napoca, Romania; steliana.ghibu@umfcluj.ro

**Keywords:** trastuzumab emtansine, trastuzumab deruxtecan, antibody-drug conjugates, HER2-positive breast cancer, HER2-targeted therapy, adverse reactions, pharmacovigilance, EudraVigilance, disproportionality analysis, descriptive analysis

## Abstract

Trastuzumab (T) and tyrosine kinase inhibitors (TKIs) are among the first-line treatments recommended for HER2-positive breast cancer. More recently, antibody-drug conjugates (ADCs) such as trastuzumab deruxtecan (T-DXd) and trastuzumab emtansine (T-DM1) have been authorized, and they represent the second-line therapy in this type of cancer. The present study aimed to evaluate adverse drug reactions (ADRs) associated with T-based ADCs that were spontaneously reported in EudraVigilance—the European pharmacovigilance database. Out of 42,272 ADRs reported for currently approved ADCs on the market, 24% of ADRs were related to T-DM1, while 12% of ADRs were related to T-DXd. T-DM1 had a higher probability of reporting eye, ear and labyrinth, and cardiac and hepatobiliary ADRs, while T-DXd had a higher probability of reporting respiratory, thoracic and mediastinal, blood and lymphatic system, metabolism and nutrition, and gastrointestinal ADRs. The present research found that in terms of hematological disorders, T-DM1 and T-DXd had a higher probability of reporting ADRs than TKIs. Moreover, the data showed that T-DM1 seemed to have a higher risk of cardiotoxicity than T-DXd, while T-DXd had a higher probability of reporting metabolism and nutrition disorders than T-DM1.

## 1. Introduction

Cancer is a widely occurring disease that causes abnormal and uncontrolled growth of cells, thus being a life-threatening condition. The conventional antineoplastic treatment commonly involves chemotherapeutic agents whose use is limited by their low specificity regarding tumor cells that can result in systemic toxicity, a narrow therapeutic window and even drug resistance [1,2].

Antibody-drug conjugates (ADCs) are monoclonal antibodies (mAbs) that are tied to a cytotoxic drug (payload) by using a chemical linker. These molecules present targeted specificity, which is correlated with fewer side effects and a wider therapeutic window when compared to the conventional cancer therapy [1,2].

Though the benefits of ADCs are numerous, their toxicity cannot be overlooked. It can be explained by the fact that part of the administered ADC does not reach the targeted cells, thus affecting non-targeted healthy ones. Although in most cases the toxicity is derived from their payload, ADCs can also bind to target antigens that are present in healthy cells, thus determining a toxic response [3].

**Figure 1:**
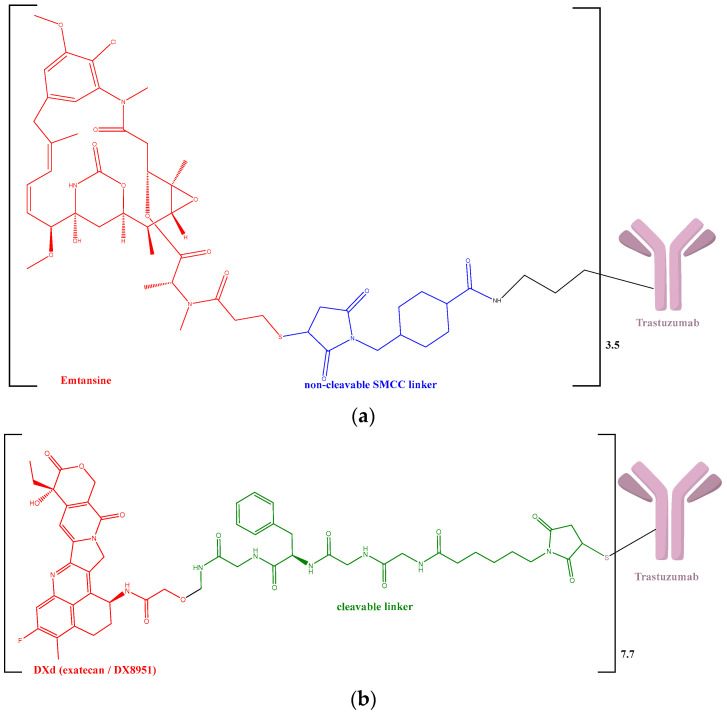
Structure formula: (**a**) trastuzumab emtansine [6,7]; (**b**) trastuzumab deruxtecan [8,9,10,11] DXd—DX8951 derivative, SMCC—Succinimidyl 4-(N-maleimidomethyl)cyclohexane-1-carboxylate.

According to the actual recommendations, mAbs (trastuzumab (T) and pertuzumab (PER)) represent the first-line treatment of the human epidermal growth factor receptor 2 (HER2)-positive tumors. These are associated with docetaxel or other taxans (paclitaxel or nab-paclitaxel). Also, capecitabine or vinorelbine may be considered when taxans are contraindicated [4]. Lapatinib (LAP), a tyrosine kinase inhibitor against the epithelial growth factor receptor 1 (EGFR1) and HER2, which inhibits the signaling pathways down from the HER2 level [5], could be recommended alone or in association with T, in case the patient is not suitable for first-line chemotherapy or for endocrine therapy. Trastuzumab deruxtecan (T-DXd) or trastuzumab emtansine (T-DM1) (Figure 1) is preferred as second-line therapy after progression on taxans and trastuzumab. Not least, other HER2-selective tyrosine kinase inhibitors (tucatinib (TUC) and neratinib(NER)) are treatment options for third-line and beyond [4]. 

T is a humanized IgG1 mAb that stops the cell cycle due to the protein kinase B phosphorylation inhibition process and it activates the antibody-dependent cell-mediated cytotoxicity process against HER2-overexpressed breast cancer tumors [12]. A recent meta-analysis confirmed that T increases the risk of infections, gastrointestinal effects or skin disorders [13], and side effects that can affect the patient’s quality of life.

Another humanized recombinant mAb, namely PER, blocks the dimerization of HER2, thus inhibiting the classical HER2-mediated cell-signaling cascades. LAP, NER, and TUC are inhibitors of tyrosine kinases (TKI) that target HER2 and inhibit the signaling pathways down from HER2 level [14,15,16,17,18].

**Figure 2:**
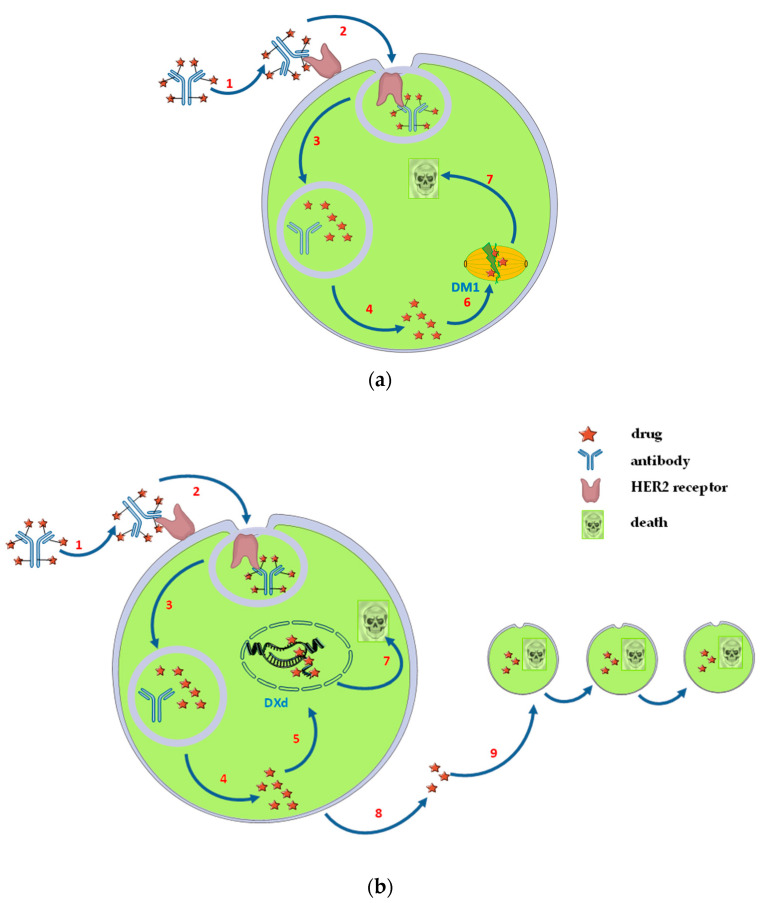
Mechanism of action: (**a**) trastuzumab emtansine; (**b**) trastuzumab deruxtecan [26,27,28]. 1—binding of the ADC to the target antigen; 2—internalization of ADC—antigen complex; 3—degradation of the cytotoxic drug inside the lysosome; 4—releasing the free cytotoxic drug; 5—destruction of DNA promoted by Dxd, a topoisomerase I inhibitor; 6—inhibition of the assembly of microtubules by binding DM1 to tubulin; 7—cell death; 8—diffusion of the free cytotoxic drug outside of targeted cell; 9—bystander effect; DM1—emtansine; DXd—DX8951 derivative.

Regarding ADCs including T, emtansine (DM1) is the payload component from T-DM1 that targets microtubules and induces death in proliferative cells by blocking cellular growth and stopping cell division (mitosis) [19,20] (Figure 2a). On the other hand, DX8951 derivative (DXd), the T-DXd payload drug, is a potent topoisomerase I inhibitor [21,22,23] (Figure 2b). T-DM1 has low payload membrane permeability and is bound by a thioether non-cleavable linker, while T-DXd has high payload membrane permeability and is bound by a cleavable tetrapeptide-based linker. Between the two, only T-DXd presents the bystander effect [24] that leads to cell death of the neighboring tumor cells by amplifying the activity of the cytotoxic drug [25] (Figure 2b).

Regarding their efficiency, a study performed by Cortes et al. showed that the risk of disease progression or death was reduced in patients treated with T-DXd compared to T-DM1 [29]. According to the results of the DESTINY-Breast03 study, patients treated with T-DXd had a significant improvement in overall survival but also a higher risk of interstitial lung disease or pneumonitis compared to T-DM1 [30].

Because structural differences between the two ADCs impart different levels of efficiency and safety, more studies are needed on this issue. The present study aims to compare the safety profile of ADCs based on T (T-DM1 and T-DXd) to other molecules administered in HER2-positive breast cancer: T, PER, LAP, NER, and TUC. Also, data for evaluated drugs were compared to data for ADCs. Not the least, data for both ADCs were compared with each other.

## 2. Materials and Methods

### 2.1. Data Source and Extraction Criteria

An evaluation of the safety profile of trastuzumab-based ADCs was performed. The ICSRs (Individual Case Safety Reports) containing T-based ADCs submitted in the EudraVigilance (EV) database were analyzed. Data uploaded until 25 February 2024 in the https://www.adrreports.eu/ platform (accessed on 26 February 2024) were extracted for ADCs (Table 1), T, PER, LAP, NER, and TUC.

### 2.2. Data Analysis

#### 2.2.1. ADRs Reported for ADCs in EV

Based on the ICSRs submitted in EV, the distribution of ADRs recorded for ADCs was calculated. Subsequently, the ratio of the total number of ADRs to the total number of ICSRs was calculated for each drug and for the entire class.

#### 2.2.2. Descriptive Analysis of ADRs Related to T-DM1 and T-DXd

A descriptive analysis of ICSRs reported in the EV database for T-DM1 and T-DXd was performed. Data were compared with those extracted for the group of other ADCs and for T, PER, LAP, TUC, and NER.

Demographic characteristics of patients were analyzed (age categories and sex). According to EMA recommendations, data collected were grouped into eight age categories (not specified, 0–1 month, 2 months–2 years, 3–11 years, 12–17 years, 18–64 years, 65–85 years, and more than 85 years) and into three categories by sex (female, male, and not specified). Other data used in the general evaluation were referred to the geographical origin (European Economic Area—EEA, non-EEA, and not specified) and reporter’s category (healthcare professional, non-healthcare professional, and not specified). Additionally, the comparison between T-DM1 and T-DXd with HER2-targeted drugs (T, PER, LAP, NER, and TUC) and ADCs group, respectively, was performed according to the severity of the case (serious, non-serious, and not specified).

#### 2.2.3. Disproportionality Analysis

According to the Medical Dictionary for Regulatory Activities (MedDRA) terminology, ADRs are reported as preferred terms grouped into “High-Level Group Terms” (HLGTs) and are linked by anatomy, pathology, physiology, etiology, or function. There are a total of 27 “System Organ Classes” (SOCs) containing HLGTs grouped by different characteristics such as etiology, manifestation site, purpose, etc. Based on SOC classification, a disproportionality analysis was performed between T-DM1 or T-DXd and HER2-targeted drugs and the group of other ADCs. Subsequently, a comparison was performed between both T-based ADCs (T-DM1 and T-DXd) to evaluate their similarities and differences related to the reporting probability for all ADRs tested. To establish the disproportionate signal, reporting odds ratio (ROR) and 95% confidence intervals (CI) [34] were calculated with MedCalc Software Ltd. Odds ratio calculator [35]; https://www.medcalc.org/calc/odds_ratio.php (Version 20.123), accessed on 26 February 2024. EMA recommends that data from EV could be analyzed if ICSRs are classified into four categories and two dichotomous variables (a two-by-two contingency table) [36]. According to recommendations, ROR calculation should be performed using comparators from common therapeutic areas [35,37]. Also, a signal was considered disproportionate if the number of cases was at least 5 and the lower limit of the 95% CI was greater than 1 [38].

### 2.3. Ethics

No ethical approval was necessary as all data were reported anonymously, and no personal data could be identified on the EV portal.

## 3. Results

### 3.1. ADRs Reported for ADCs in EV

**Figure 3:**
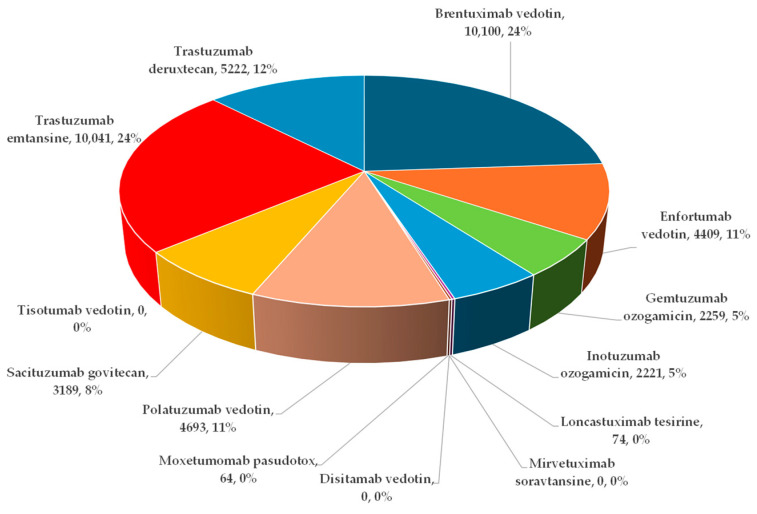
Distribution of ADRs reported in EV for ADCs.

A total number of 42,272 ADRs were reported in 21,515 ICSRs submitted to EV until 25 February 2024. The highest number of ADRs was registered for bretuximab vedotin (BV, n = 10,100; 24%), T-DM1 (n = 10,041; 24%), and T-DXd (n = 5222; 12%). No ADRs were reported in EV for mirvetuximab soravtansine and tisotumab vedotin (Figure 3).

**Figure 4:**
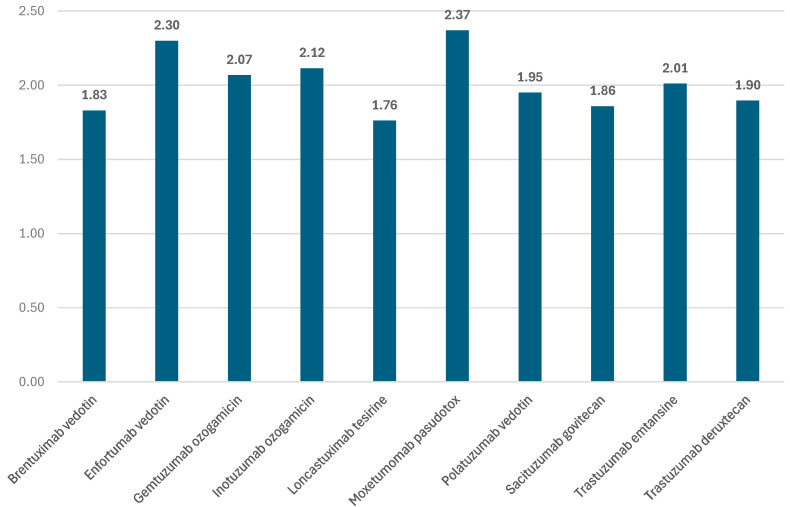
Proportion of total ADRs to total number of ICSRs for each analyzed ADC.

The mean ratio of total ADRs to total ICSRs reported in EV was 2.02 (minimum: 1.76 for loncastuximab tesirine and maximum: 2.37 for moxetumomab pasudotox). For T-DM1 and T-DXd, the ratios were close to the average value (2.01 and 1.90) (Figure 4).

### 3.2. Descriptive Analysis of ICSRs Reported in the EV Database for Trastuzumab, Trastuzumab Emtansine, and Trastuzumab Deruxtecan

The distribution of ICSRs reported for T-DM1 and T-DXd according to patient demographics (age category and sex) is presented in Table 1 compared to the other HER2-targeted drugs (T, PER, LAP, TUC, and NER) and the group of other ADCs.

Regarding the age category, similar proportions were observed for all age categories, except the 18–64 years category and the not specified category. A higher proportion compared to T-DM1 (47.38%) and T-DXd (34.51%) was registered for T (51.89%) and PER (55.37%). Also, in the 18–64 years and 65–85 years categories, the reports for the other ADCs have high proportions (34.97 and 30.23%, respectively). In the class of TKIs, the majority of reports were registered in the 18–64 years category for LAP (48.31%) and NER (46.15%). Also, no notable differences were observed in sex categories between different TKIs. The situation was different for T-DM1 due to the elevated proportion of cases reported for the female group (93.69%). For T-DM1, the indications are limited to breast cancer, compared to T (breast cancer and gastric cancer) and T-DXd (breast cancer, pulmonary cancer, and gastric cancer). Also, PER and TKIs present the same overwhelmingly female prevailing trend. For example, the cases reported in the female group have the following proportions: PER (92.88%), LAP (93.23%), TUC (92.81%), and NER (92.84%). But, for the other ADCs, the distribution of ICSRs by sex (female—40.81% and male—48.11%) differs from the one observed in T-DM1 or T-DXd (Table 2).

Table 3 presents the distribution of ICSRs by geographical origin (EEA, non-EEA, and not specified) and reporter category (healthcare professionals—HP, non-healthcare professionals—non-HP, and not specified—NS). T-DM1 and T-DXd had a similar proportion of ICSRs reported from the EEA to other comparators except TKIs (T-DM1—38.45%, T-DXd—38.98%, other ADCs—34.57%, T—38.01%, and PER—35.90%). Also, regarding the proportion of HP that submitted ICSRs, small differences were observed between T-DM1 (90.55%) or T-DXd (94.44%) and T (91.44%), PER (90.96%), and other ADCs (95.84%). Reports including TKIs that were submitted by HP had a lower frequency (LAP—83.04%, TUC—73.10%, and NER—85.41%).

**Figure 5:**
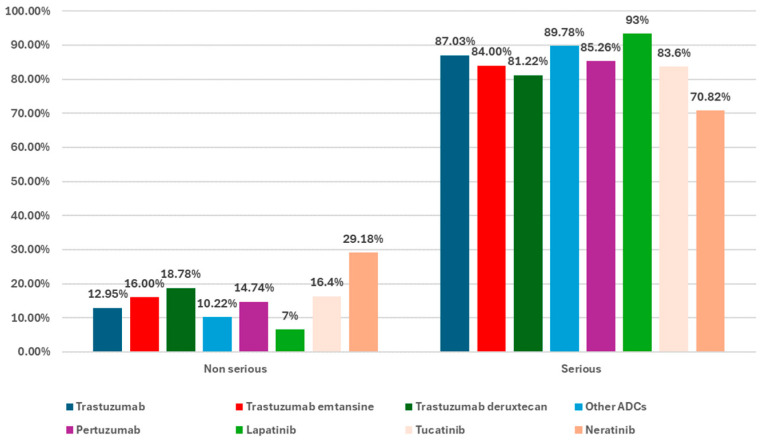
The seriousness of cases reported in EV.

Figure 5 presents the severity of the cases reported in EV for T-DM1 and T-DXd compared to HER2-targeted drugs or other ADCs. The percentage of ICSRs reported as serious compared to non-serious ICSRs was higher for all drugs. However, the proportion of serious cases for T-DXd (81.22%) and T-DM1 (84.00%) was slightly lower compared to T (87.03%), PER (85.26%), and other ADCs (89.78%). Only in 7 ICSRs for T (0.02%) the seriousness of cases was not specified. An interesting observation was remarked upon TKIs: LAP had the highest proportion of serious cases (93.00%) and NER the lowest (70.82%) among all drugs analyzed.

### 3.3. Disproportionality Analysis

#### 3.3.1. Probability of Reporting ADRs Related to T-DM1

**Figure 6:**
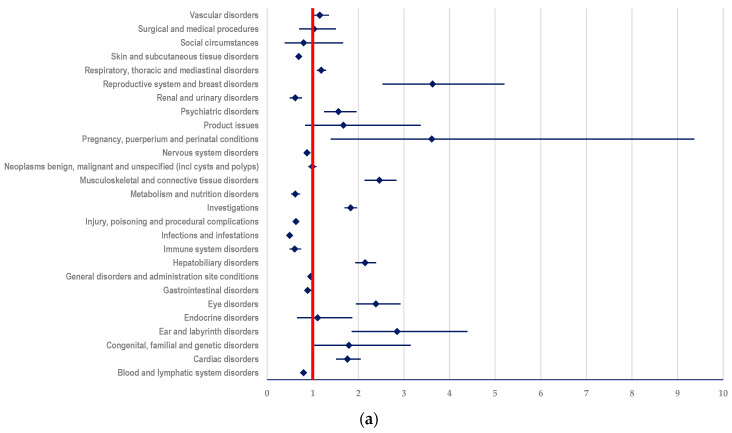

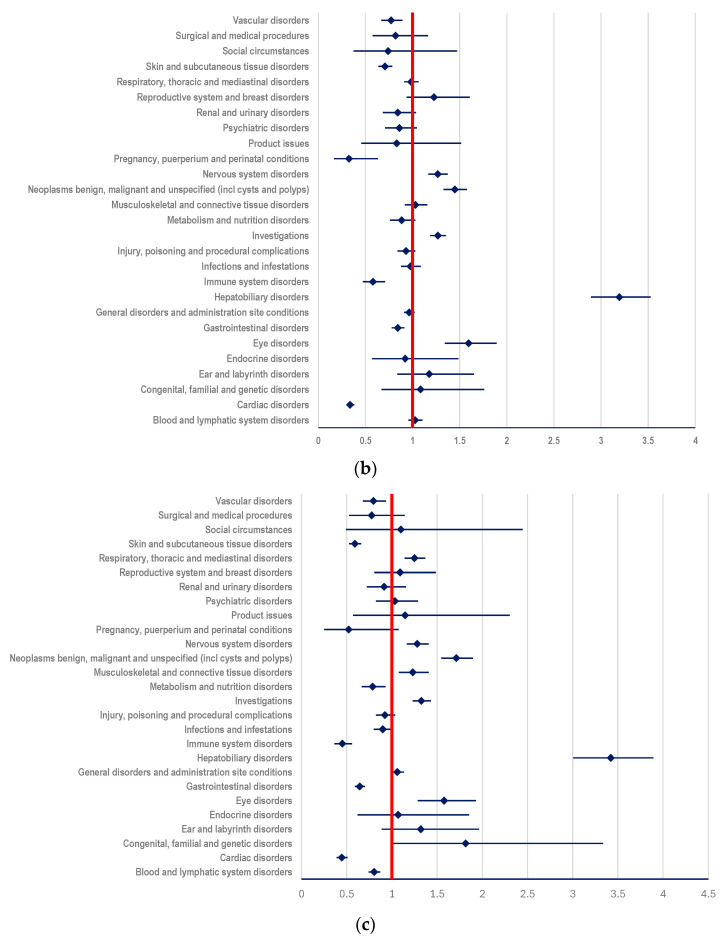

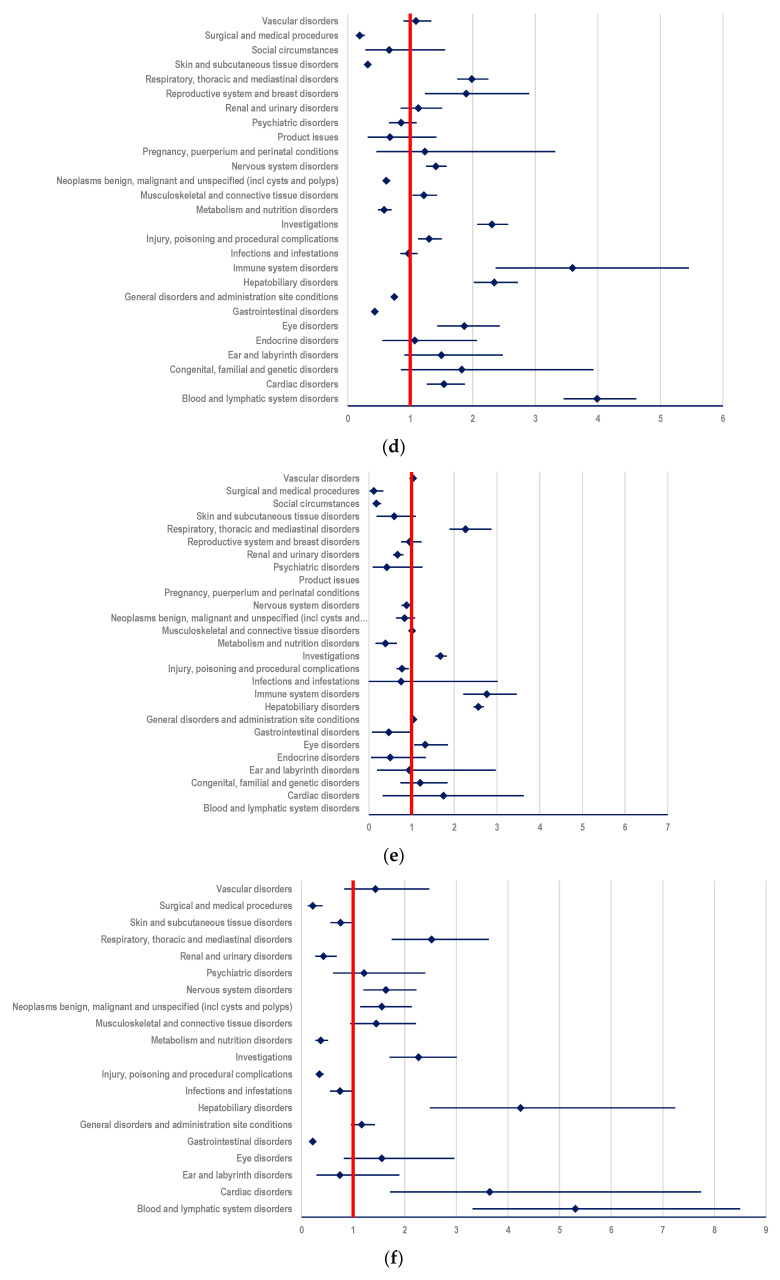
Reporting odds ratio of ADRs related to T-DM1: (**a**) T-DM1 vs. other ADCs; (**b**) T-DM1 vs. T; (**c**) T-DM1 vs. PER; (**d**) T-DM1 vs. LAP; (**e**) T-DM1 vs. TUC; (**f**) T-DM1 vs. NER.

ADRs related to T-DM1 and included in SOC “Hepatobiliary disorders” had a higher reporting probability than all other comparators:

(i) ADCs (ROR: 2.1496; 95% CI: 1.9338-2.3894) (Figure 6a);
(ii) mAbs: T (ROR: 3.1958; 95% CI: 2.8956-3.5270) and PER (ROR: 3.4217; 95% CI: 3.0069-3.8938) (Figure 6b,c)
(iii) TKIs: LAP (ROR: 2.3419; 95% CI: 2.0155-2.7212), TUC (ROR: 2.5647; 95% CI: 2.0131-3.2676), and NER (ROR: 4.2442; 95% CI: 2.4878-7.2408) (Figure 6d–f).

Compared to TKIs, T-DM1 had a disproportionate signal for ADRs included in SOC “Blood and lymphatic system disorders” (LAP: ROR-3.9904; 95% CI: 3.4514-4.6138; TUC: ROR-5.8665; 95% CI: 4.4454-7.7419; NER: ROR-5.3046; 95% CI: 3.3113-8.4980). Similar results were observed in SOC “Cardiac disorders” (LAP: ROR-1.5382; 95% CI: 1.2634-1.8728; TUC: ROR-1.7460; 95% CI: 1.2801-2.3813; NER: ROR-3.6437; 95% CI: 1.7150-7.7415) (Figure 6d, 6e, and 6f). Also, for these categories of ADRs, T-DM1 showed a higher reporting probability compared to the group of other ADCs (ROR-1.7611; 95% CI: 1.5126-2.0503) (Figure 6a).

ADRs included in SOC “Eye disorders” were reported with a higher probability for T-DM1 than for other ADCs (ROR: 2.3860; 95% CI: 1.9454-2.9265) (Figure 6a) and mAbs (T-ROR: 1.5953; 95% CI: 1.3437-1.8940 and PER-ROR: 1.5770; 95% CI: 1.2873-1.9319) (Figure 6b,c).

Compared to other ADCs (ROR: 2.8524; 95% CI: 1.8524-4.3920) (Figure 6a), T-DM1 showed a disproportionate signal for ADRs included in SOC “Ear and labyrinth disorders”.

In SOC “Musculoskeletal and connective tissue disorders”, T-DM1 had a disproportionate signal compared to other ADCs (ROR: 2.4630; 95% CI: 2.1380-2.8373) (Figure 6a), PER (ROR: 1.2303; 95% CI: 1.0762-1.4064) (Figure 6c), and LAP (ROR: 1.2160; 95% CI: 1.0350-1.4286) (Figure 6d).

Compared to mAbs (T-ROR: 1.2672; 95% CI: 1.1689-1.3736 and PER-ROR: 1.281; 95% CI: 1.1670-1.4061) (Figure 6b,c) and TKIs (LAP-ROR: 1.4085; 95% CI: 1.2548-1.5810 and NER-ROR: 1.6349; 95% CI: 1.2010-2.2255) (Figure 6d,f), ADRs from SOC “Nervous system disorders” related to T-DM1 had a higher reporting probability.

Except for T, the probability of reporting ADR from SOC “Respiratory, thoracic and mediastinal disorders” was higher for T-DM1:

(i) Other ADCs: ROR: 1.1880; 95% CI: 1.0909-1.2938 (Figure 6a)
(ii) PER: ROR: 1.2496; 95% CI: 1.1408-1.3687 (Figure 6c)
(iii) LAP: ROR: 1.9838; 95% CI: 1.7512-2.2474 (Figure 6d)
(iv) TUC: ROR: 2.2591; 95% CI: 1.8482-2.7613 (Figure 6e)
(v) NER: ROR: 2.5178; 95% CI: 1.7474-3.6278 (Figure 6f)

#### 3.3.2. Probability of Reporting ADRs Related to T-DXd

**Figure 7:**
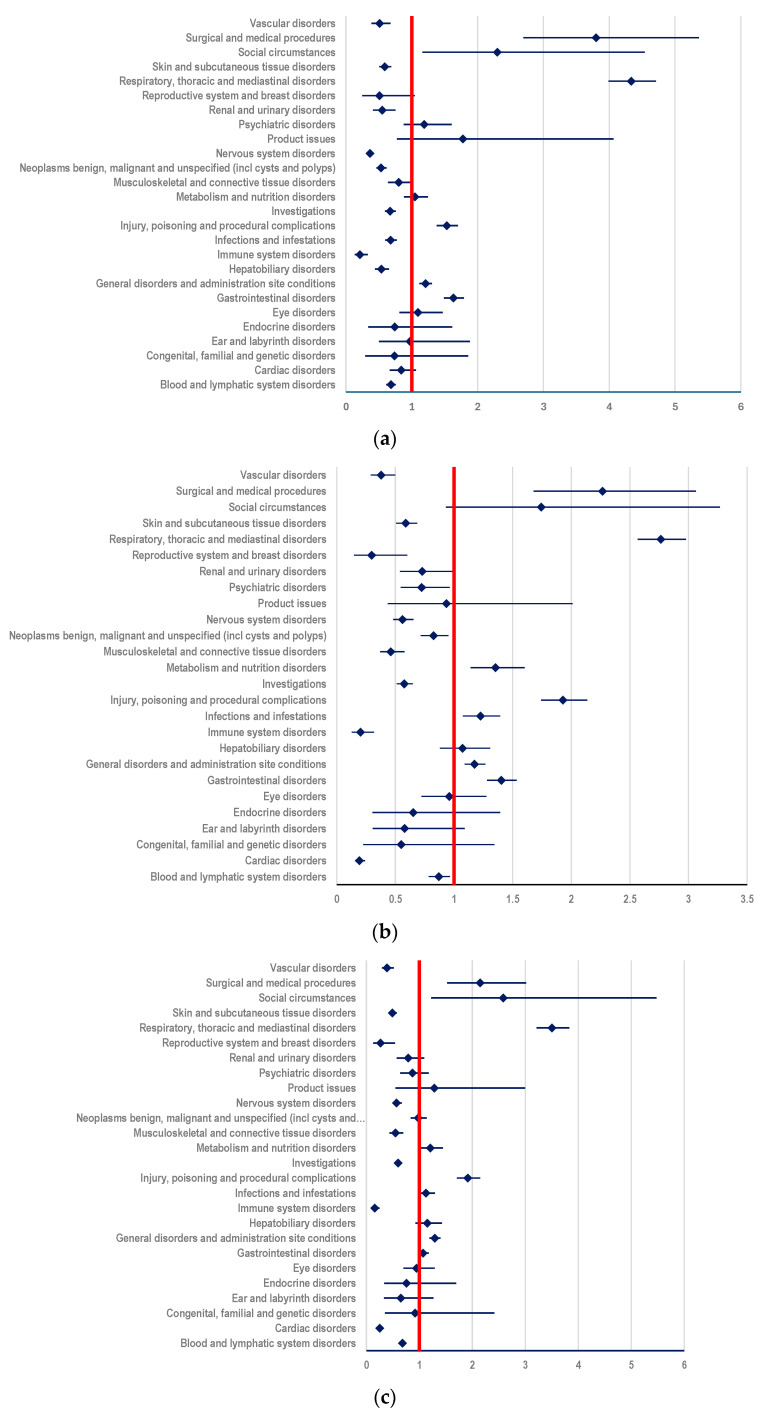

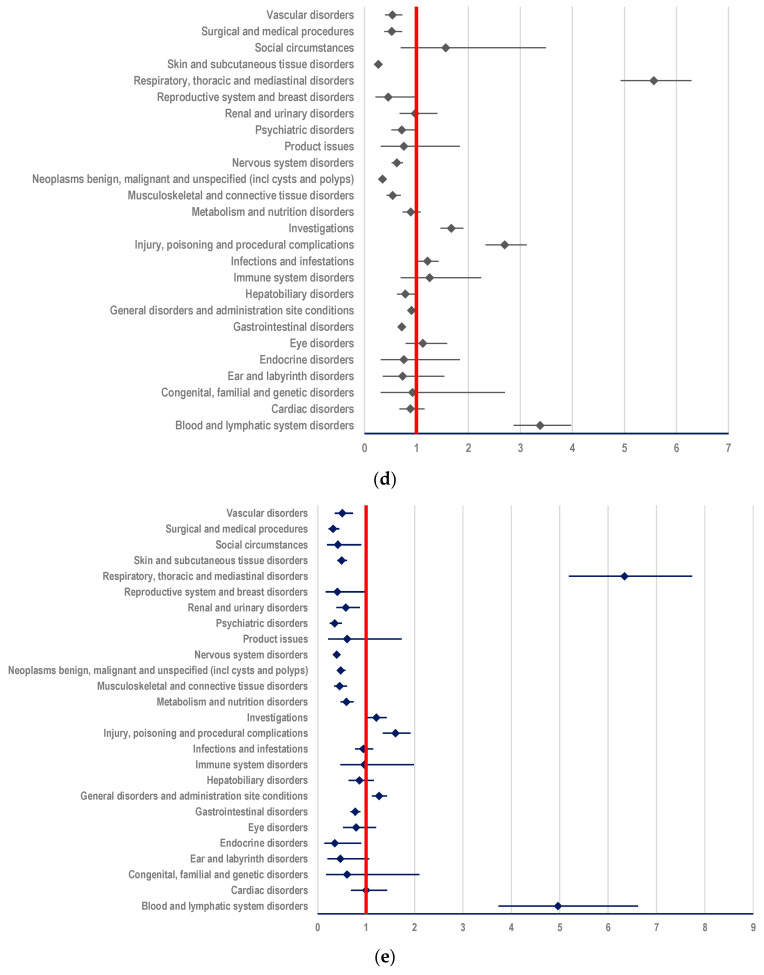

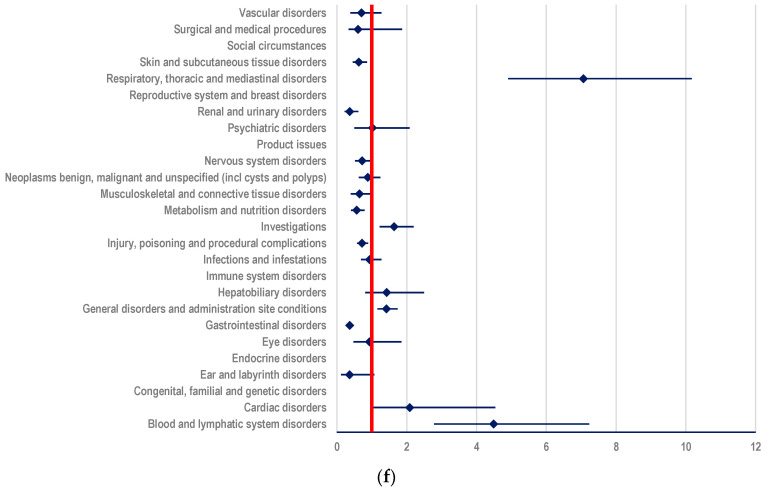
Reporting odds ratio of ADRs related to T-DXd: (**a**) T-DXd vs. other ADCs; (**b**) T-DXd vs. T; (**c**) T-DXd vs. PER; (**d**) T-DXd vs. LAP; (**e**) T-DXd vs. TUC; (**f**) T-DXd vs. NER.

According to our results, a higher probability of reporting ADRs related to T-DXd was observed for several SOCs. For example, a disproportionate signal was observed for ADRs included in SOC “Respiratory, thoracic and mediastinal disorders” compared to:

(i) other ADCs: ROR-4.3343; 95% CI: 3.9893-4.7091 (Figure 7a);
(ii) mAbs: T: ROR-2.7646; 95% CI: 2.5669-2.9776; PER: ROR-3.5059; 95% CI: 3.2112-3.8276 (Figure 7b,c);
(iii) TKIs: LAP: ROR-5.5659; 95% CI: 4.9248-6.2905; TUC: ROR-6.3382; 95% CI: 5.1930-7.7360; NER: ROR-7.0641; 95% CI 4.9065-10.1703 (Figure 7d–f);

Also, in SOC “Blood and lymphatic system disorders” a disproportionate signal was observed compared to TKIs:

(i) LAP: ROR-3.3774, 95% CI: 2.8693-3.9755 (Figure 7d);
(ii) TUC: ROR-4.9652, 95% CI: 3.7258-6.6169 (Figure 7e);
(iii) NER: ROR-4.4896; 95% CI: 2.7862-7.2345 (Figure 7f).

Compared to mAbs, the probability of reporting ADRs was also higher in SOC “Metabolism and nutrition disorders” (PER: ROR-1.2037; 95% CI: 1.0027-1.4450; T: ROR-1.3535; 95% CI: 1.1430-1.6028) (Figure 7b,c).

Last but not least, ADRs included in SOC “Gastrointestinal disorders” had a higher reporting probability compared to T (ROR: 1.4030; 95% CI: 1.2834-1.5337) (Figure 7b) and other ADCs (ROR-1.6327; 95% CI: 1.4868-1.7930) (Figure 7a).

#### 3.3.3. Comparison between T-DM1 and T-DXd

**Figure 8:**
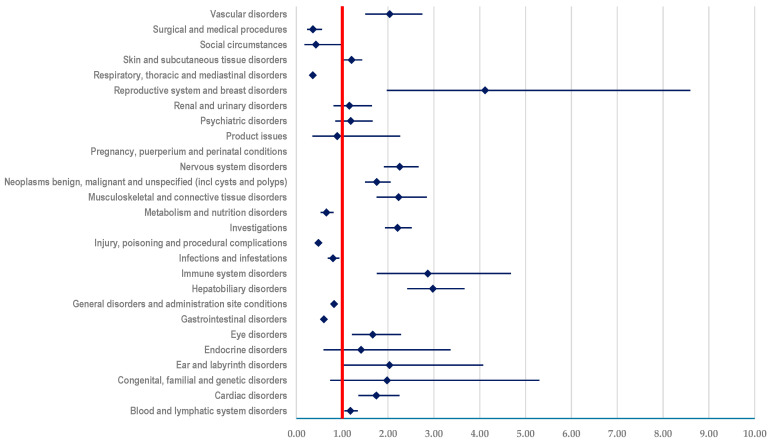
Reporting odds ratio of ADRs related to T-DM1 compared to T-DXd.

By comparing the two studied ADCs, it was observed that the probability of reporting an ADR included in the following SOCs is higher for T-DM1 than for T-DXd: (i) “Blood and lymphatic system disorders” (ROR: 1.1815, 95% CI: 1.0467-1.3370), (ii) “Cardiac disorders” (ROR: 1.7442, 95% CI: 1.3532-2.2482) or (iii) “Vascular disorders” (ROR: 2.0344, 95% CI: 1.5055-2.7491). Also, a higher probability of reporting was observed for T-DM1 compared to T-DXd for ADRs included in SOC “Musculoskeletal and connective tissue disorders” (ROR: 2.2304; 95% CI: 1.7489-2.8443), SOC “Neoplasms benign, malignant and unspecified (incl cysts and polyps)” (ROR: 1.7534; 95% CI: 1.4961-2.0548), and SOC “Nervous system disorders” (ROR: 2.2546; 95% CI: 1.9091-2.6625) (Figure 8).

Furthermore, our study showed that T-DM1 had a lower reporting probability for ADRs included in the following SOCs: “Gastrointestinal disorders” (ROR: 0.6015; 95% CI: 0.5368-0.6740), “Metabolism and nutrition disorders” (ROR: 0.6537; 95% CI: 0.5271-0.8107), “Respiratory, thoracic and mediastinal disorders” (ROR: 0.3564; 95% CI: 0.3223-0.3941) when compared to T-DXd (Figure 8).

## 4. Discussion

ADCs are an expanding drug class with a positive benefit-to-risk ratio for cancer patients. Being relatively new on the market, their safety and efficacy represent topics of interest for the health system [3].

T marked a new era in cancer treatment, targeting the HER2-positive breast tumor, a particularly difficult and aggressive form of neoplasm. At a subsequent time, T-DM1 and T-DXd, two ADCs containing T, were developed to better showcase the advantages of the molecule and to diminish the disadvantages [39]. The present research is focused on spontaneous reports of ADRs related to T-DM1 and T-DXd filed in EV, undertaking a comprehensive context analysis in comparison to the other approved ADCs, T itself, other drugs prescribed for HER2-positive breast cancer, and to each other.

In the group of ADCs, BV had the highest number of reports in EV. Several factors may explain this top position, such as (i) the relatively long time from first marketing authorization, (ii) the introduction of the drug in prescription protocols, (iii) patient supervision by trained medical personnel, and (iv) efficient counseling to empower the patient to self-identity adverse reactions. BV is prescribed in adults for the treatment of Hodgkin lymphoma (HL), CD30+ HL, systemic anaplastic large cell lymphoma, and cutaneous T-cell lymphoma [40,41,42]. Its presence in therapy for more than a decade has proven its efficacy, with the drug currently being considered a component of standard treatment for patients with advanced disease [43], however, peripheral neurotoxicity was observed and reported, arising concerns and triggering attentive patient monitoring [44]. The survival rate of patients treated with BV was higher compared to that of patients following other protocols, and the neurotoxicity was less marked after time elapsed from the last administration [45].

Among ADCs, T-DM1 is positioned as the second highest in terms of the number of recorded ADRs, at a short distance from BV. Each of the two accounted for approximately 24% of the total ADRs. T-DXd was third, at a considerable distance, with 12% (Figure 3). Recent data evaluated by Liu et al. regarding T-DM1 and T-DXd ADRs from the FDA Adverse Event Reporting System (FAERS), another relevant database, is consistent with the higher trend of reports for T-DM1 compared to T-DXd. However, two major differences should be noted; first, the substantially lower total number of ADRs reported for T-DM1 and T-DXd in that database, and second, the distinct rough ratio of reports T-DM1:T-DXd which was 3:2 [46].

Taking into consideration the guidelines’ recommendations for breast cancer therapy, the results linking T, T-DM1, and T-DXd to an elevated proportion of cases reported for adult women were expected and confirmed (Table 2). Although rare, breast cancer may affect men [47,48]. The presence of numerous reports, observed for the ADCs of interest, filed by non-EEA-located health professionals, suggests an elevated level of trust, prestige, and access to EV.

Compared to the group of other ADCs, T-DM1 has a higher probability of reporting for several categories such as “Eye disorders”, “Ear and labyrinth disorders”, “Cardiac disorders”, “Hepatobiliary disorders”, etc. Various research groups have documented eye disorders in patients under treatment with T-DM1, such as corneal changes [49,50], e.g., cystoid lesions in the deep corneal epithelial cells [51] and lacrimal drainage system stenosis [52]. Ocular toxicity of T-DM1 also had a high frequency in a pharmacovigilance study conducted by Liu et al. on FAERS database [46]. A different FAERS pharmacovigilance study, which only took into account cardiovascular adverse events, found that T-DM1 had the lowest signal in heart failure and cardiomyopathy, while T had the highest one, and no significant signal was observed in T-DXd [53]. Pondé et al. suggest that T-DM1-associated cardiotoxicity is actually rare, but it could mean discontinuing the treatment [54]. The EMA review of T-DM1 for the adjuvant treatment of adult patients with HER2-positive early breast cancer found that hepatotoxicity is among the major risks associated with T-DM1 [55]. Increased gamma-glutamyltransferase was among the most common effects of T-DM1 in a phase IIIb study [56]. Also, a case study from France has identified an unusual liver toxicity event in a T-DM1 patient [57].

In SOC “Blood and lymphatic system disorders”, T-DM1 and T-DXd have a higher probability of reporting ADR compared to TKIs. In the DESTINY-Breast03 study of T-DXd, the most severe but not life-threatening adverse effects occurring in patients receiving T-DXd were hematological, such as neutropenia, thrombocytopenia, and leukopenia [58]. This was also shown in a pharmacovigilance study consulting the FAERS database [46] and in a meta-analysis of randomized controlled trials [59]. Regarding T-DM1, an EMA review identified hemorrhage and thrombocytopenia as major risks [55]. A phase IIIB study has shown that the incidence of thrombocytopenia after treatment with T-DM1 is higher in Asian populations [60].

Compared to mAbs, the probability of reporting ADR was higher in the category “Metabolism and nutrition disorders” for T-DXd, but not for T-DM1. 

Regarding the “Gastrointestinal disorders”, our study showed only a higher probability of reporting for T-DXd compared to T and the group of other ADCs. Also, our study is in accordance with the DESTINY-Breast03 study, showing that the gastrointestinal adverse effects (namely nausea and vomiting) were more prevalent for T-DXd than T-DM1 [58]. Similar results were obtained in the pharmacovigilance study consulting the FAERS database [46]. Other studies comparing the use of T-DXd and T-DM1 in metastatic breast cancer [30] and breast cancer [29] found that nausea and vomiting were the most frequent adverse events for T-DXd.

Due to the disproportionate signal resulting by comparison to reference drugs, T-DM1 seems to have a higher risk of “Cardiac disorders” than T-DXd. A pharmacovigilance study consulting the FAERS database concluded that the cardiotoxicity of both T-DM1 and T-DXd is lower than that of T; however, T-DM1 did show a higher proportion of cardiotoxic adverse effects than T-DXd [46]. As stated earlier, a cardiotoxicity focused FAERS study found that T-DXd had a lower risk than T-DM1 as well [53]. A systematic review found that T-DXd treatment has a low incidence of cardiotoxicity [61]. However, there have been cases of unexpected T-DXd cardiotoxicity reported [62].

Our study shows that both T-DM1 and T-DXd have a higher probability of reporting ADRs from SOC “Respiratory, thoracic and mediastinal disorders”. In the DESTINY-Breast03 study of T-DXd, 10.5% of patients that received T-DXd developed lung diseases, as opposed to 1.9% of patients that received T-DM1 [58]. Moreover, the monitoring of the pulmonary events is recommended in patients treated with T-DXd [29,30]. The pharmacovigilance study consulting the FAERS database results also showed a higher frequency of severe pulmonary adverse effects in the case of T-DXd than T-DM1, whereas T-DM1 showed a high epistaxis frequency [46]. A phase two study, a meta-analysis of randomized controlled trials, and a systematic review also found that interstitial lung disease/pneumonia were among the risks involved with the use of T-DXd [59,61,63]. However, most of these events can be managed with careful monitoring and prompt intervention [64].

Both ADCs do not present a disproportionate signal for some SOCs compared to all drugs (“Renal and urinary disorders”, “Skin and subcutaneous tissue disorders”, “Vascular disorders”). A study comparing T-DM1 to T combined with paclitaxel found no significant difference in the incidence of skin disorder adverse events [65]. But, a meta-analysis found a potential skin necrosis risk when T-DM1 is combined with radiotherapy [66]. The same results were obtained for “Psychiatric disorders” except for T-DM1 when compared to other ADCs. Thus, a lower risk of the occurrence of ADRs from these SOCs could be expected.

### Limitations of the Study

Safety analysis based on large pharmacovigilance databases containing real-world data has typical limitations, which include the phenomenon of under-reporting, over-reporting, and reporting bias. Due to their spontaneous nature, the data included in the ICSRs may be incomplete or of poor quality, often lacking information on clinical characteristics, medical status, concomitant medication, comorbidities, outcome, follow-up, etc. Also, the present study only provides an overview of adverse drug reactions that have been reported, the real number of patients receiving the analyzed drugs not being known. Interpretation of these results requires caution, given the lack of a denominator. A particularity of pharmacovigilance in oncology is represented by the fact that targeted therapies are considered to have fewer adverse reactions and thus they may be underestimated and consequently underreported, especially by healthcare professionals [67]. Thus, the descriptive and disproportionality analysis performed does not quantify the actual risk of adverse drug reactions, providing only an identification of a safety signal. Lastly, a causal relationship between the drugs analyzed and the reported ADRs cannot be established, and further studies are needed to evaluate their safety profile.

## 5. Conclusions

Antibody-drug conjugates represent promising therapeutics in oncology. For HER2-positive breast cancer, two examples of the above-mentioned class are trastuzumab deruxtecan and trastuzumab emtansine. This study focused on evaluating the adverse reactions recorded in EudraVigilance for T-DM1 and T-DXd. Correlations were made with reports for other relevant comparison items such as ADCs, several drugs prescribed for HER2-positive breast cancer (T, PER, LAP, NER, TUC), and each other. The number of ADR reports for T-DM1 was double that for T-DXd, the latter being more recently approved on the market. Compared to the group of other ADCs, T-DM1 has a higher probability of reporting eye, ear and labyrinth, and cardiac and hepatobiliary ADRs, while T-DXd has a higher probability for respiratory, thoracic and mediastinal, and gastrointestinal ADRs. Both T-DM1 and T-DXd have a higher probability of reporting hematological disorders’ ADRs than TKIs do. The results suggest a higher risk of cardiotoxicity for T-MD1 than for T-DXd, but T-DXd prevails over T-DM1 in the probability of reporting metabolism and nutrition disorders. Safety and efficacy evaluation is important for realistic drug profiling. Further research should be conducted for a thorough profile of such complex molecules.

## Figures and Tables

**Table 1:** ADCs approved on the market [31,32,33].

ADC	Year of Approval on the Market	Indication	Reports Registered in the EV Database
Gemtuzumab ozogamicin *	2000	acute myelogenous leukaemia	Yes
Brentuximab vedotin	2011	Hodgkin lymphoma	Yes
Trastuzumab emtansine	2013	HER2-positive breast cancer	Yes
Inotuzumab ozogamicin	2017	acute lymphoblastic leukaemia	Yes
Moxetumomab pasudotox	2018	hairy cell leukaemia	Yes
Enfortumab vedotin	2019	metastatic urothelial cancer	Yes
Polatuzumab vedotin	2019	beta-cell lymphoma	Yes
Trastuzumab deruxtecan	2019	HER2-positive breast cancer	Yes
Sacituzumab govitecan	2020	triple-negative breast cancer	Yes
Tisotumab vedotin	2021	cervical cancer	No
Disitamab vedotin	2021	advanced breast cancer	No
Loncastuximab tesirine	2021	beta-cell lymphoma	Yes
Mirvetuximab soravtansine	2022	ovarian cancer	No

* Withdrawn from the market in 2010 and reapproved in 2017.

**Table 2:** The comparison of demographic data from EV for patients treated with T-DM1 or T-DXs and the other ADCs or other drugs used in HER2-positive breast cancer.

		T-DXd n (%)	T-DM1 n (%)	Other ADCs n (%)	T n (%)	PER n (%)	LAP n (%)	TUC n (%)	NER n (%)
**Total ICSR**		2753	4994	13,768	37,461	11,452	4268	1015	377
**Age category**	Not specified	1350	1823	3996	10,722	3063	1576	649	155
		(49.04)	(36.50)	(29.02)	(28.62)	(26.75)	(36.93)	(63.94)	(41.11)
	0–1 month	0	0	2	16	2	0	2	0
		(0.00)	(0.00)	(0.01)	(0.04)	(0.02)	(0.00)	(0.20)	(0.00)
	2 months–2 years	3	1	18	16	3	0	0	0
		(0.11)	(0.02)	(0.13)	(0.04)	(0.03)	(0.00)	(0.00)	(0.00)
	3–11 years	0	0	122	4	1	3	0	0
		(0.00)	(0.00)	(0.89)	(0.01)	(0.01)	(0.07)	(0.00)	(0.00)
	12–17 years	0	0	185	6	1	3	0	0
		(0.00)	(0.00)	(1.34)	(0.02)	(0.01)	(0.07)	(0.00)	(0.00)
	18–64 years	950	2366	4814	19,439	6341	2062	269	174
		(34.51)	(47.38)	(34.97)	(51.89)	(55.37)	(48.31)	(26.50)	(46.15)
	65–85 years	433	769	4162	6994	1980	598	95	48
		(15.73)	(15.40)	(30.23)	(18.67)	(17.29)	(14.01)	(9.36)	(12.73)
	More than 85 years	17	35	469	264	61	26	0	0
		(0.62)	(0.70)	(3.41)	(0.70)	(0.53)	(0.61)	(0.00)	(0.00)
**Sex**	Female	2469	4679	5619	33,081	10,637	3979	942	350
		(89.68)	(93.69)	(40.81)	(88.31)	(92.88)	(93.23)	(92.81)	(92.84)
	Male	214	68	6624	2491	246	118	28	11
		(7.77)	(1.36)	(48.11)	(6.65)	(2.15)	(2.76)	(2.76)	(2.92)
	Not specified	70	247	1525	1889	569	171	45	16
		(2.54)	(4.95)	(11.08)	(5.04)	(4.97)	(4.01)	(4.43)	(4.24)

n—number of reports.

**Table 3:** Distribution of ICSRs by geographical origin and reporter category.

		T-DM1 n (%)	T-DXd n (%)	Other ADCs n (%)	T n (%)	PER n (%)	LAP n (%)	TUC n (%)	NER n (%)
**Geographical origin of the reporter**	EA	1920	1073	4760	14,238	4111	1118	293	180
		(38.45)	(38.98)	(34.57)	(38.01)	(35.90)	(26.19)	(28.87)	(47.75)
	NON-EEA	3074	1680	9008	23,223	7341	3150	722	197
		(61.55)	(61.02)	(65.43)	(61.99)	(64.10)	(73.81)	(71.13)	(52.25)
**Reporter** **Category**	HP	4522	2600	13,195	34,256	10,417	3544	742	322
		(90.55)	(94.44)	(95.84)	(91.44)	(90.96)	(83.04)	(73.10)	(85.41)
	Non-HP	472	153	572	3182	1035	724	273	55
		(9.45)	(5.56)	(4.15)	(8.49)	(9.04)	(16.96)	(26.90)	(14.59)
	NS	0	0	1	23	0	0	0	0
		(0.00)	(0.00)	(0.01)	(0.06)	(0.00)	(0.00)	(0.00)	(0.00)

n—number of reports; EEA—European Economic Area, NON-EEA—non-European Economic.

## Data Availability

Data are contained within the article.
